# Parents’ Knowledge, Attitude, and Practice Related to Childhood Constipation in Makkah, Saudi Arabia

**DOI:** 10.7759/cureus.52236

**Published:** 2024-01-13

**Authors:** Albraa J Khayyat, Refal T Abumansour, Moath A Khayat, Nada O Almalayo, Raghad E Saleh, Doaa S Baashar, Musaad M Almhmadi, Rayan O Almalki, Mohammed Ageel

**Affiliations:** 1 Department of Medicine and Surgery, College of Medicine, Umm Al-Qura University, Makkah, SAU; 2 Department of Medicine and Surgery, College of Medicine, Umm Al-Qura University, makkah, SAU; 3 Department of Surgery, Faculty of Medicine, Umm Al-Qura University, Makkah, SAU

**Keywords:** saudi arabia, practice, parents, knowledge, constipation, childhood, attitude

## Abstract

Introduction

Functional constipation in children is described as irregular or difficult bowel motions without underlying systemic or anatomical causes. Although constipation can have a serious negative impact on a child's health and the lives of their parents. This study aimed to assess the knowledge of parents about childhood constipation, intending to reduce morbidity and mortality through increased public health education in Makkah, Saudi Arabia.

Methods

The current study was a web-based, descriptive cross-sectional study. The data were obtained from May 2023 to November 2023 through an online questionnaire directed to parents in Makkah, Saudi Arabia, and analyzed using SPSS version 27.0.1 (Armonk, NY: IBM Corp.).

Results

A total of 796 participants were included in the present study, of which 205 (25.8%) were males and 591 (74.2%) were females. The knowledge levels among them varied, with 11.1% correctly defining constipation and 63.6% recognizing it as a symptom. Common causes like organic and functional constipation were acknowledged by 88.4% and 81.3% of participants, respectively. Regarding practices, 27.9% recommended high-fiber foods for initial home treatment, and 42.8% acknowledged that an enema is effective for fecal impaction. In the dietary recommendations, 71.7% suggested fruits and 68.8% mentioned vegetables. Concerning attitudes, 65.1% expressed fear of childhood constipation continuing into adulthood, while 30.9% feared severe medical conditions. The internet (25.5%) and friends/relatives (23.6%) were the primary sources of information. Knowledge was significantly higher among females and those with postgraduate education.

Conclusion

This study highlights the variability in knowledge levels among parents, with an overall moderate understanding of childhood constipation. It emphasizes a moderate level of adherence to recommended practices related to childhood constipation, with some room for improvement in Makkah, Saudi Arabia.

## Introduction

Constipation can notably affect children's health and their parents' quality of life. Functional constipation (FC) in children is defined as irregular or complex bowel movements without underlying systemic or anatomical causes [1]. Although the prevalence of constipation varies among societies based on many factors, including sociocultural and dietary habits, constipation is a common pediatric health problem, affecting between 0.7% and 29.6% of children [2]. One study reported inadequate nutrition as a primary (58%) risk factor for constipation, with mental instability (21.2%) and genetic factors (3.5%) also identified [3].

Worldwide, many diagnostic criteria have been approved for diagnosing functional constipation. However, the Rome III criteria and the North American Society for Pediatric Gastroenterology, Hepatology, and Nutrition (NASPGHAN) guidelines are the clinical criteria most often utilized in Saudi Arabia [4]. Recently, the Rome IV criteria have been widely used to diagnose FC. According to Rome IV, the diagnosis of FC in children under the age of four requires the existence of at least two of the following symptoms for at least one month in the absence of an organic cause and with insufficient criteria to diagnose irritable bowel syndrome: maximum of two attempts to defecate per week, history of constipation, history of uncomfortable, painful bowel movements, history of hard or large stools, having a large fecal mass in the rectum. Rome IV also notes the following criteria for diagnosing FC in toilet-trained children over four years of age: one or more incontinence incidences per week and a history of having large, thick stools that could block the toilet [5].

Fecal incontinence, frequent abdominal pain, bleeding of the rectum, enuresis, and urinary retention and infection are all well-recognized complications of FC in children [6]. Fecal occlusion, intestinal/ileus blockage, toxic megacolon, and bowel perforation are the more catastrophic potential effects of FC [7]. The North American and European Societies for Pediatric Gastroenterology, Hepatology, and Nutrition (NASPEGHAN/ESPGHAN) released updated clinical recommendations for diagnosing and managing FC in children. These include "a normal intake of fibers and liquids, normal physical activity, and an extra pharmacological treatment for rectal fecal impaction followed by a pharmacological maintenance therapy." The societies also recommend polyethylene glycol (PEG) with or without electrolytes (0.2-0.8 g/kg) for maintenance therapy [8].

Providentially, FC is estimated to account for 90% of cases seen in healthcare centers, with the remaining 5-10% having organic causes [9]. Because FC is a relatively benign disorder, it often goes undiagnosed and untreated, which can lead to a variety of medical and psychosocial issues for children and their parents, cause concern for healthcare budgets, and, presumably, place a significant socioeconomic burden [1,10,11]. A 2022 study in Saudi Arabia found that parents' knowledge of childhood constipation was linked to their practices in dealing with it. The study involved 568 participants and concluded that poor practices were due to inadequate knowledge [12]. A 2019 study in kindergartens in Jatinangor found that in 111 parents there was a correlation between parental knowledge and their children's toilet training practice and behavior. The study highlights the importance of educating parents about fecal continence in children [13]. In addition, a 2020 systematic review of 13 studies attempted to locate, organize, and synthesize the current data on the experiences of parents caring for children with FC and the information needed by such parents to facilitate successful treatment. The study revealed that most parents have inadequate knowledge of childhood constipation, with a relatively small percentage having sufficient and correct information about the condition [14].

In this study, pediatricians were also found to have inadequate knowledge of FC in children, with notable differences in the pediatricians' knowledge and patterns of practice regarding childhood constipation [3,4,14]. To the best of our knowledge, no studies in the Western region of Saudi Arabia assess the level of parents' knowledge regarding childhood FC. Thus, the study aimed to evaluate parents' knowledge, attitudes, and practices regarding childhood FC in Makkah, Saudi Arabia to identify knowledge gaps that can be filled to reduce morbidity through early detection, appropriate access to medical care, and increased public awareness.

## Materials and methods

Study design and participants

This web-based descriptive cross-sectional study evaluated parents' knowledge, attitude, and practice toward childhood constipation. The data were obtained through an online questionnaire directed to parents in Makkah, Saudi Arabia. Parents working in the medical field were excluded.

Ethical considerations and sampling technique

The researchers were committed to all ethical considerations required to conduct the research. The Biomedical Ethics Committee at Umm Al-Qura University provided ethical approval no. HAPO-02-K-012-2023-04-1539 to conduct this study. Participants’ privacy was maintained, and the responses remained confidential. Using the Raosoft sample size calculator (Seattle, WA: Raosoft, Inc.), the sample size was determined with a 95% confidence level, a 5% margin of error, and a 50% response distribution, and 385 participants were considered the minimal sample size [2]. The overall sample size increased to a maximum of 824 participants in case of potential data loss and to generalize the study results more efficiently.

Study tool and data collection

A previously validated questionnaire has been utilized [12]. An online questionnaire was sent electronically through Google Forms to 824 parents; two opted out of the study and 26 were related to the health sector. After the exclusion of these 28 parents, a total of 796 parents satisfying the inclusion criteria were included in the analysis. The questionnaire is composed of three sections. The first section comprised parents' sociodemographic characteristics, such as age, gender, employment status, residence, and educational status. The second part included seven questions to evaluate parents' knowledge, causes, and symptoms. The third section assessed the response, treatment, and source of knowledge. The questionnaire has been closed-ended with predefined choices. The questionnaire was already prepared and translated into Arabic, the local language. Google Forms have been used for the design.

Statistical analysis

Descriptive and inferential statistical analyses of the data were carried out. Simple descriptive statistics of the sociodemographic characteristics and other categorical variables in the form of frequencies and percentages were calculated and tabulated. Continuous variables medians and interquartile ranges (IQRs) were reported as measures of central tendency and dispersion, respectively, owing to the relatively non-normal distribution of variables as determined by the Kolmogorov-Smirnov test (p<0.001). Seven questions assessed the parents' knowledge and awareness of childhood constipation, and one point was given for each correct response. These were summed up to obtain the total knowledge score of each participant. Because some questions involved multiple responses, the total possible knowledge score of a participant ranged from 0 to 18. Similarly, the practice scores of the participants ranged from 0 to 4. The scores of participants with different sociodemographic characteristics were compared using the non-parametric Mann-Whitney U test and the Kruskal-Wallis test. Additionally, non-parametric Spearman's rank correlation was applied to assess the correlation between knowledge and practice scores. Significance was established at a p-value of 0.05, indicating a 95% confidence interval. All statistical calculations were performed using SPSS version 27.0.1 (Armonk, NY: IBM Corp.).

## Results

Among 796 participants, a notable majority were female, accounting for 74.2% of the sample, while the remaining 25.8% were male. Each participant willingly agreed to partake in the survey. The age distribution varied, with the largest group falling in the 35-60 years category at 62.8%, followed by 25 to 34-year-olds at 22.0%; a smaller representation of individuals aged 15-24 years (6.7%) and those above 60 years (8.5%). The educational backgrounds of the participants were diverse, with a significant majority having a university education (67.5%), while 12.1% possessing postgraduate qualifications. The participants also came from various occupational backgrounds, with government employees making up the largest group (34.3%) and unemployed individuals accounting for 31.0%. Most participants were married (92.5%), while 7.5% were divorced. Additionally, 80.7% of the participants reported that their children had previously experienced constipation (Table 1).

**Table 1:** Sociodemographic characteristics of the participants.

Characteristics		N	%
Gender	Female	591	74.2%
	Male	205	25.8%
Age	15-24 years	53	6.7%
	25-34 years	175	22.0%
	35-60 years	500	62.8%
	More than 60 years	68	8.5%
Educational level	Middle	21	2.6%
	Nothing	2	0.3%
	Postgraduate	96	12.1%
	Primary	9	1.1%
	Secondary	131	16.5%
	University education	537	67.5%
Occupation	An employee in the private sector	85	10.7%
	Self-employed	45	5.7%
	Government employee	273	34.3%
	Retired	146	18.3%
	Unemployed	247	31.0%
Marital status	Divorced	60	7.5%
	Married	736	92.5%
Have any of your children suffered from constipation before?	No	154	19.3%
	Yes	642	80.7%

Table 2 presents the participants’ responses to knowledge questions regarding childhood constipation, highlighting the percentages of correct answers. Only 11.1% of participants correctly identified the definition of constipation as “less than three bowel movements per week,” while 63.6% correctly recognized it as a “symptom” rather than an illness. In terms of common causes, a significant 88.4% recognized “organic constipation” and 81.3% identified “functional constipation” as common causes. Regarding the causes of functional constipation in children, 84.3% correctly attributed it to a “low-fiber diet.” In terms of symptoms, substantial percentages correctly identified “hard, dry stool” (71.0%) and “painful defecation” (63.0%) as common symptoms. However, fewer participants recognized the symptom of “large diameter stool that may obstruct the toilet” (19.3%). Concerning the necessity of complete tests for constipated children, 38.7% correctly answered “no,” while 30.2% believed “yes” and 31.2% responded with “I don't know.” Finally, for complications, the majority correctly identified “painful anal fissures” (75.1%) and “fecal impaction” (60.6%) as potential complications, while fewer recognized “intestinal perforation” (7.3%) and “fecal incontinence” (8.3%). The participants’ total knowledge score had a median of 8.0, indicating a moderate overall understanding of childhood constipation. This summary emphasizes the varying levels of correct answers among the participants and underscores areas where educational efforts may be beneficial (Table 2).

**Table 2:** Responses of the participants to knowledge questions. *Correct answer.

Characteristics		N	%
Among the following, which is closest to the definition of constipation?	Difficulty in excretion	398	50.0%
	Lack of daily output	173	21.7%
	Less than three bowel movements per week*	88	11.1%
	Metaphyseal ossification (stool)	137	17.2%
In your opinion, is constipation a disease or a symptom?	A symptom*	506	63.6%
	I don't know	102	12.8%
	Illness	188	23.6%
What is the common cause of constipation in children (multi-select question)?	Organic constipation*	704	88.4%
	Functional constipation*	647	81.3%
	I don't know	101	12.7%
What is the cause of functional constipation in children (multi-select question)?	Stool withholding*	267	36.1%
	Toilet training resistance*	147	19.9%
	Low-fibers diet*	623	84.3%
	Low physical activity*	252	34.1%
	I don't know	78	10.6%
What are the most common symptoms of constipation (multi-select question)?	Abdominal pain*	390	50.1%
	Painful defecation*	491	63.0%
	Hard, dry stool*	553	71.0%
	Large diameter stool that may obstruct the toilet*	150	19.3%
Does a constipated child need complete tests at all times?	I don't know	248	31.2%
	No*	308	38.7%
	Yes	240	30.2%
What are the complications of constipation in children (multi-select question)?	Painful anal fissures (torn skin around the anus)*	598	75.1%
	Rectal prolapse (intestine that protrudes from the anus)*	216	27.1%
	Fecal impaction (stool that can't be expelled)*	482	60.6%
	Fecal incontinence (leaking of watery stool from the bottom)*	66	8.3%
	Intestinal perforation*	58	7.3%
Total knowledge score - median (IQR)		8.0 (6.0-10.0)	

Table 3 provides insights into the participants’ responses regarding their practices related to childhood constipation, with a focus on the percentages of correct practices. When asked about the initial home treatment for their child’s constipation, 27.9% correctly suggested “giving him high-fiber food,” while 23.5% recognized the importance of “giving him plenty of fluids.” However, a notable 23.7% mentioned “giving him laxatives” as an initial treatment, which is not recommended without medical guidance. Furthermore, only a small percentage (10.6%) suggested “give him bananas or honey.” Regarding the treatment for fecal impaction and intestinal blockage, 42.8% correctly identified “do an enema to expel stool” as the most successful treatment, indicating a good understanding of this critical aspect. In terms of recommending fiber-rich foods for children with constipation, 71.7% rightly suggested “fruits like watermelon, apple, and banana” and 68.8% recognized “vegetables” as a suitable option. This indicates a relatively strong awareness of dietary recommendations for managing constipation. The participants’ total practice score had a median of 2.0 (IQR: 1.0-3.0), suggesting a moderate level of adherence to correct practices related to childhood constipation (Table 3).

**Table 3:** Responses of the participants to practice questions. *Correct practice.

Variables		N	%
What is the initial treatment that you do at home to treat your child’s constipation before taking him to the doctor?	Give him bananas or honey	84	10.6%
	Give him laxatives	189	23.7%
	Giving him high-fiber food*	222	27.9%
	Giving him plenty of fluids	187	23.5%
	Massage	72	9.0%
	Warm bath	42	5.3%
In the event of fecal impaction and blockage of the intestine, in your opinion, what is the most successful treatment?	Do an enema to expel stool*	341	42.8%
	Give him laxatives	292	36.7%
	High-fiber foods	117	14.7%
	Manual emptying of rectal contents by a doctor	46	5.8%
From the list below, what is the fiber-rich food that you recommend giving to a child with constipation (multi-select question)?	Rice	53	6.7%
	Fruit like watermelon, apple, and banana*	571	71.7%
	Bread	45	5.7%
	Vegetable*	548	68.8%
	Potatoes	55	6.9%
Practice score - median (IQR)		2.0 (1.0-3.0)	

When asked about their biggest concern regarding chronic childhood constipation, the majority (65.1%) expressed the fear of it “continuing into adulthood.” A significant portion also expressed concerns related to severe medical conditions, with 30.9% fearing “internal abdominal tumors” and 17.3% fearing “congenital abnormalities of the colon (stricture).” These responses highlight the substantial apprehension parents may have about the long-term implications of childhood constipation on their children’s health (Table 4).

**Table 4:** Responses of the participants to attitude questions.

Variables		N	%
What is your biggest fear from chronic childhood constipation (multi-select question)?	The fear of being from congenital abnormalities of the colon (stricture)	138	17.3%
	The fear of being from internal abdominal tumors	246	30.9%
	The fear of continuing to adulthood	518	65.1%
What is your source of information about constipation?	Doctors and medical staff	116	14.6%
	Frequent medical practice	110	13.8%
	Friends and relatives	188	23.6%
	Internet	203	25.5%
	Magazines and books	40	5.0%
	Other	108	13.6%
	TV	31	3.9%

**Figure 1:**
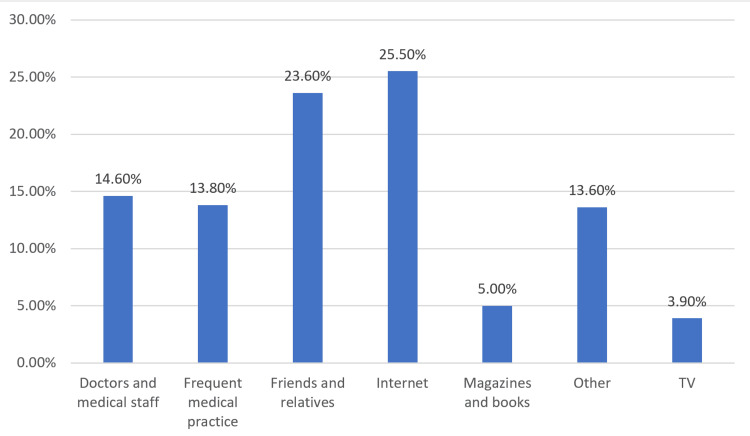
Source of information of the participants.

Regarding sources of information about constipation, the internet emerged as the most commonly cited source, with 25.5% of the participants relying on online resources. Friends and relatives also played a significant role, with 23.6% turning to them for information. Doctors and medical staff were mentioned by 14.6% of the participants, while frequent medical practice was cited by 13.8%. Magazines, books, TV, and other sources made up the remainder of the information channels. This indicates a diverse range of sources from which parents seek information about childhood constipation, with the internet being a prominent choice. This underscores the importance of ensuring accurate and reliable online resources for parents seeking guidance on this health issue (Figure 1).

The findings indicated a significant difference in knowledge scores based on gender (p<0.001). Female participants had a higher median knowledge score of 8.0 (IQR: 7.0-10.0) compared to males, with a median score of 8.0 (IQR: 5.0-10.0). Regarding educational level, there was a significant difference in knowledge scores (p=0.001). Postgraduate participants exhibited the highest median knowledge score of 9.0 (IQR: 7.0-11.0), while those with a middle-level education had the lowest median score of 7.0 (IQR: 5.0-9.0). However, no significant associations were observed between knowledge scores and age, occupation, marital status, or whether their children had experienced constipation before.

In summary, parents and educational level were associated with variations in knowledge about childhood constipation among the participants. Female participants and those with postgraduate education tended to have higher knowledge scores (Table 5).

**Table 5:** Association of knowledge score with sociodemographic characteristics. ^*^Independent samples Mann-Whitney U test. ^**^Independent samples Kruskal-Wallis test. ^***^P-value <0.05 was considered significant.

Variables		Knowledge score		p-Value^*,**^
		Median	IQR	
Gender	Female	8.0	7.0-10.0	<0.001***
	Male	8.0	5.0-10.0	
Do you agree to participate in the survey?	Yes, I agree	8.0	6.0-10.0	
Age	0-24 years	8.0	5.0-10.0	0.764
	25-34 years	8.0	6.0-10.0	
	35-60 years	8.0	6.0-10.0	
	More than 60 years	8.0	6.0-10.0	
Educational level	Middle	7.0	5.0-9.0	0.001***
	Nothing	7.5	5.0-10.0	
	Postgraduate	9.0	7.0-11.0	
	Primary	7.0	6.0-7.0	
	Secondary	7.0	5.0-10.0	
	University education	8.0	7.0-10.0	
Occupation	An employee in the private sector	8.0	6.0-10.0	0.562
	Self-employed	8.0	5.0-10.0	
	Government employee	8.0	6.0-11.0	
	Retired	8.0	6.0-10.0	
	Unemployed	8.0	6.0-10.0	
Marital status	Divorced	8.0	5.5-10.0	0.470
	Married	8.0	6.0-10.0	
Have any of your children suffered from constipation before?	No	8.0	6.0-10.0	0.162
	Yes	8.0	6.0-10.0	

The analysis revealed some significant findings. First, there was a significant difference in practice scores based on age (p=0.007). The participants in the 25-34 years age group had a median practice score of 2.0 (IQR: 1.0-3.0; mean=2.0), and those in the 35-60 years group (mean=2.2) and more than 60 years age group (mean=2.4) also had a median score of 2.0 (IQR: 2.0-3.0). However, participants in the 15-24 years age group had a comparatively lower score of 2.0 (IQR: 1.0-3.0) (mean=1.9). Other sociodemographic characteristics, including gender, agreement to participate in the survey, educational level, occupation, marital status, and whether their children had experienced constipation before, did not show significant associations with practice scores (Table 6).

**Table 6:** Association of practice score with sociodemographic characteristics. ^*^Independent samples Mann-Whitney U test. ^**^Independent samples Kruskal-Wallis test. ^***^P-value <0.05 was considered significant.

Variables		Practice score		p-Value^*,**^
		Median	IQR	
Gender	Female	2.0	2.0-3.0	0.320
	Male	2.0	1.0-3.0	
Do you agree to participate in the survey?	Yes, I agree	2.0	1.0-3.0	
Age	15-24 years	2.0	1.0-3.0	0.007***
	25-34 years	2.0	1.0-3.0	
	35-60 years	2.0	2.0-3.0	
	More than 60 years	2.0	2.0-3.0	
Educational level	Middle	2.0	2.0-3.0	0.191
	Nothing	1.0	0.0-2.0	
	Postgraduate	2.0	2.0-3.0	
	Primary	2.0	0.0-2.0	
	Secondary	2.0	1.0-3.0	
	University education	2.0	1.0-3.0	
Occupation	An employee in the private sector	2.0	1.0-3.0	0.261
	Self-employed	2.0	1.0-3.0	
	Government employee	2.0	2.0-3.0	
	Retired	2.0	2.0-3.0	
	Unemployed	2.0	1.0-3.0	
Marital status	Divorced	2.0	1.0-3.0	0.853
	Married	2.0	1.0-3.0	
Have any of your children suffered from constipation before?	No	2.0	1.0-3.0	0.288
	Yes	2.0	2.0-3.0	

**Figure 2:**
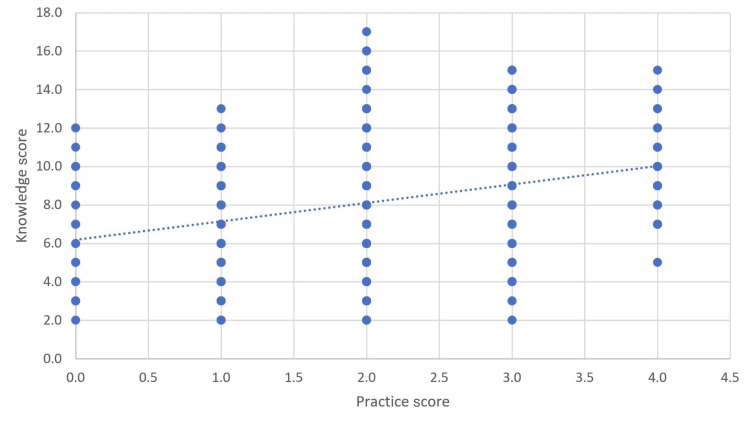
Correlation of knowledge and practice scores of the participants.

The analysis, performed using Spearman’s rank correlation coefficient, revealed a statistically significant positive correlation (ρ=0.328, p<0.001) between knowledge and practice scores among the 796 participants (Table 7). This finding indicates that the participants who demonstrated higher levels of knowledge about childhood constipation tended to exhibit better practices related to its management. In other words, as the participants’ knowledge scores increased, their practice scores also showed a positive trend. This suggests that improving knowledge about childhood constipation can lead to more effective and appropriate practices in its management and prevention (Figure 2).

**Table 7:** Correlation of knowledge and practice scores of the participants.

Variables			Practice score
Spearman's rho	Knowledge score	Correlation coefficient	0.328
		Sig. (two-tailed)	0.000
		Number of participants (n)	796

## Discussion

Our study aimed to determine parents’ knowledge, attitudes, and practices toward childhood constipation in a major city in Saudi Arabia. The dominance of female participants, constituting 74.2% of the sample, was a noteworthy observation.

Moreover, the high percentage of participants with a university education (67.5%) and postgraduate qualifications (12.1%) reflects a relatively well-educated sample. Educated parents are more likely to actively seek information and engage in health-related discussions [15]. However, the educational diversity within the sample may lead to varying levels of health literacy. This diversity highlights the importance of tailoring health education programs to different educational backgrounds to ensure effective communication and understanding.

This study provides valuable insights into the participants’ understanding of childhood constipation. While many respondents correctly identified common causes of constipation, such as “organic constipation” and “functional constipation,” there were noticeable gaps in their knowledge [1]. This indicates that constipation is perceived differently by individuals in the Makkah community. For instance, only 11.1% of persons correctly identified the definition of constipation as “less than three bowel movements per week,” consistent with the findings of Gray [16]. This lack of awareness regarding a fundamental aspect of constipation could have led to misconceptions and delayed recognition of the condition, which may have implications for children’s health. Thus, there is a lack of standardized definitions and a shared understanding of constipation within the Makkah community [17]. This lack of consensus could result in challenges in healthcare settings, as healthcare providers may need to clarify and educate patients and their families about what constipation entails.

Additionally, the relatively low recognition of specific symptoms, such as “large diameter stool that may obstruct the toilet,” and severe complications, such as “intestinal perforation,” underscores areas where parents may not be fully aware of the potential seriousness of childhood constipation, echoing concerns raised by the study conducted in Nigeria [18]. This highlights the need for comprehensive education that not only addresses common aspects of the condition but also delves into less prevalent but critical facets to ensure a holistic understanding among parents in Makkah.

The practice assessment reflects the actions that parents take when their child experiences constipation. A significant percentage suggested giving high-fiber foods and plenty of fluids as initial home treatments, which aligns with evidence-based recommendations for managing childhood constipation [19,20]. However, the finding that 23.7% of the participants mentioned “give him laxatives” as an initial treatment is concerning. This finding aligns with concerns about laxative misuse in similar populations, as identified by Roerig et al. [21]. The inappropriate use of laxatives can lead to dependence, electrolyte imbalances, and other adverse effects, highlighting the need for caution in their administration. It is crucial to highlight the importance of clear and accessible guidance on appropriate home remedies to avoid potential harm to children. Education campaigns can play a pivotal role in promoting safe and effective home treatments.

The expression of concern by parents regarding the long-term consequences of childhood constipation is a significant finding. This reflects parental anxieties about their children’s health, which has been documented by Rajindrajith et al. [22]. The fear of constipation continuing into adulthood and concerns about severe medical conditions indicate that parents in Makkah are deeply invested in their children’s well-being. This reflects a forward-looking approach to healthcare decisions. Healthcare providers should recognize and address these fears during consultations, offering reassurance and guidance to alleviate parental anxiety [23]. This personalized approach to addressing parental concerns can improve overall healthcare experiences for families in Makkah.

The analysis of associations among knowledge, practice, and sociodemographic factors revealed intriguing patterns. There was a significant difference in the knowledge scores based on gender and educational level. The female participants and those with postgraduate education tended to have higher knowledge scores. This suggests that tailored educational interventions can be particularly effective for these groups. Additionally, it highlights the importance of gender-inclusive health education efforts to bridge the knowledge gap among parents.

The positive correlation between knowledge and practice scores is a crucial finding. It suggests that when parents are better informed and have a deeper understanding of constipation, they are more likely to implement effective strategies for caring for their children with constipation. It emphasizes that improving parents’ knowledge about childhood constipation can lead directly to better practices in managing the condition, as indicated by Thompson et al. [14]. This underlines the potential impact of educational interventions in enhancing the care and well-being of children with constipation in Makkah.

The limitations of this study should be acknowledged to provide a comprehensive assessment of its findings and implications. The study’s reliance on a self-report survey may introduce response bias, as the participants may provide socially desirable answers or unintentionally misrepresent their knowledge, attitudes, and practices related to childhood constipation. The research was conducted in Makkah, Saudi Arabia, and may not be fully representative of the broader Saudi population. The predominantly female sample may also introduce gender bias and limit the generalizability of the results. Third, the study’s cross-sectional design restricts the ability to establish causal relationships between sociodemographic factors and knowledge or practice scores. Additionally, the survey’s reliance on closed-ended questions may not capture the depth of the participants’ attitudes and practices, and qualitative research methods could complement future investigations in this regard.

## Conclusions

This study highlights the variability in knowledge levels among parents, with a moderate overall understanding of childhood constipation. It emphasizes a moderate level of adherence to recommended practices related to childhood constipation, with some room for improvement in certain areas. These findings underscore the importance of targeted educational efforts to improve parents’ understanding and behavior concerning childhood constipation in Makkah, Saudi Arabia. Additionally, the study emphasizes the role of the internet and interpersonal sources in disseminating information, highlighting the need for reliable online resources and healthcare professionals to provide accurate guidance to concerned parents.
